# Elevated Factor VIII in the Setting of Recurrent Ischemic Strokes

**DOI:** 10.7759/cureus.97506

**Published:** 2025-11-22

**Authors:** Nika Bakshi, Maya S Kardouh, Claire Wang, Tanya Amal

**Affiliations:** 1 Internal Medicine, Oakland University William Beaumont School of Medicine, Royal Oak, USA; 2 Internal Medicine, Beaumont Hospital, Royal Oak, USA

**Keywords:** factor viii, ischemic stroke, lacunar infarct, lacunar infarction, tenecteplase, tia

## Abstract

We report a case of a 52-year-old female who first developed an acute lacunar infarction in the left thalamus. Two and a half years later, the patient presented with a new infarction due to high-grade stenosis of the mid basilar artery, which required treatment with tenecteplase (TNK) and angioplasty with stenting. Approximately one month following this, the patient developed a new pontine infarction despite a patent basilar artery stent. This prompted a hypercoagulable workup, which revealed an elevated factor VIII level.

## Introduction

In the United States, approximately 795,000 individuals suffer from a stroke annually [1]. Given that strokes are associated with significant morbidity and mortality, it is important that we identify risk factors for strokes to recognize and address them appropriately. Unlike the traditional risk factors such as hypertension, diabetes, and smoking, an important but often unnoticed risk factor is factor VIII [1]. Factor VIII has been linked to an increased risk of blood clots in veins and arteries by promoting thrombin formation and fibrin generation [2,3]. Despite this, it is not something that is typically checked during stroke workups. In this case report, we highlight a 52-year-old female who experienced multiple strokes over two and a half years, with her only risk factor being diabetes. Her workup eventually revealed elevated levels of factor VIII. Her case adds to growing evidence that elevated factor VIII may play a bigger role in stroke risk than we currently account for and raises the question of whether it should be considered more often in patients without a clear cause for their strokes.

## Case presentation

A 52-year-old lifetime nonsmoking female with a BMI of 23.17 kg/m^2^, blood pressure of 128/69, cholesterol of 187 mg/dL (reference range: <200 mg/dL), low-density lipoprotein of 120 mg/dL (reference range: ≤129 mg/dL), and a hemoglobin A1c of 9.5 presented with a chief complaint of right-sided weakness that began three days prior to presentation. Her neurological exam was unremarkable. Her speech was fluent without any aphasia or dysarthria. Her exam showed intact cranial nerves and 5/5 strength in all extremities with adequate tone and bulk. Her reflexes were 2/4 in the biceps, triceps, and brachioradialis and 1/4 in the patellar and Achilles reflexes. Her sensory and cerebellar exam was also unremarkable. Despite a normal neurologic exam, a magnetic resonance imaging (MRI) scan was ordered due to concerns for a stroke, since the patient mentioned her weakness had gradually improved over the course of three days.

**Figure 1:**
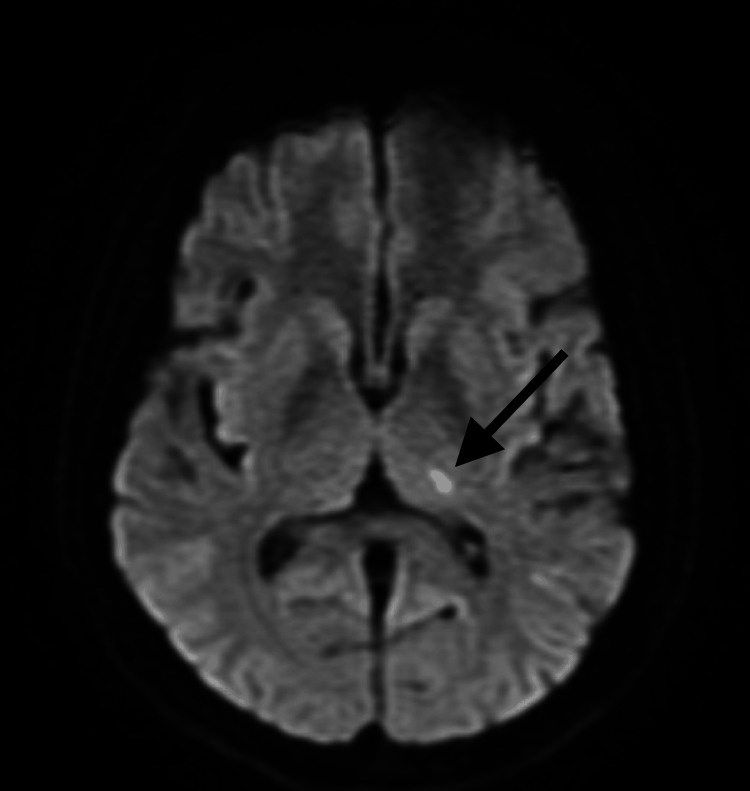
Axial diffusion-weighted imaging (DWI) MRI demonstrating an acute lacunar infarction in the left thalamus (indicated by the black arrow).

MRI confirmed an acute lacunar infarction in the left thalamus (Figure 1). An echocardiogram noted an ejection fraction of 60% and grade 1 left ventricular diastolic dysfunction and no evidence of an intracardiac thrombus or patent foramen ovale. Her carotid ultrasound demonstrated a 25-50% stenosis bilaterally. The patient was monitored and discharged home, since this small-vessel disease was likely secondary to the patient's poorly controlled diabetes.

**Figure 2:**
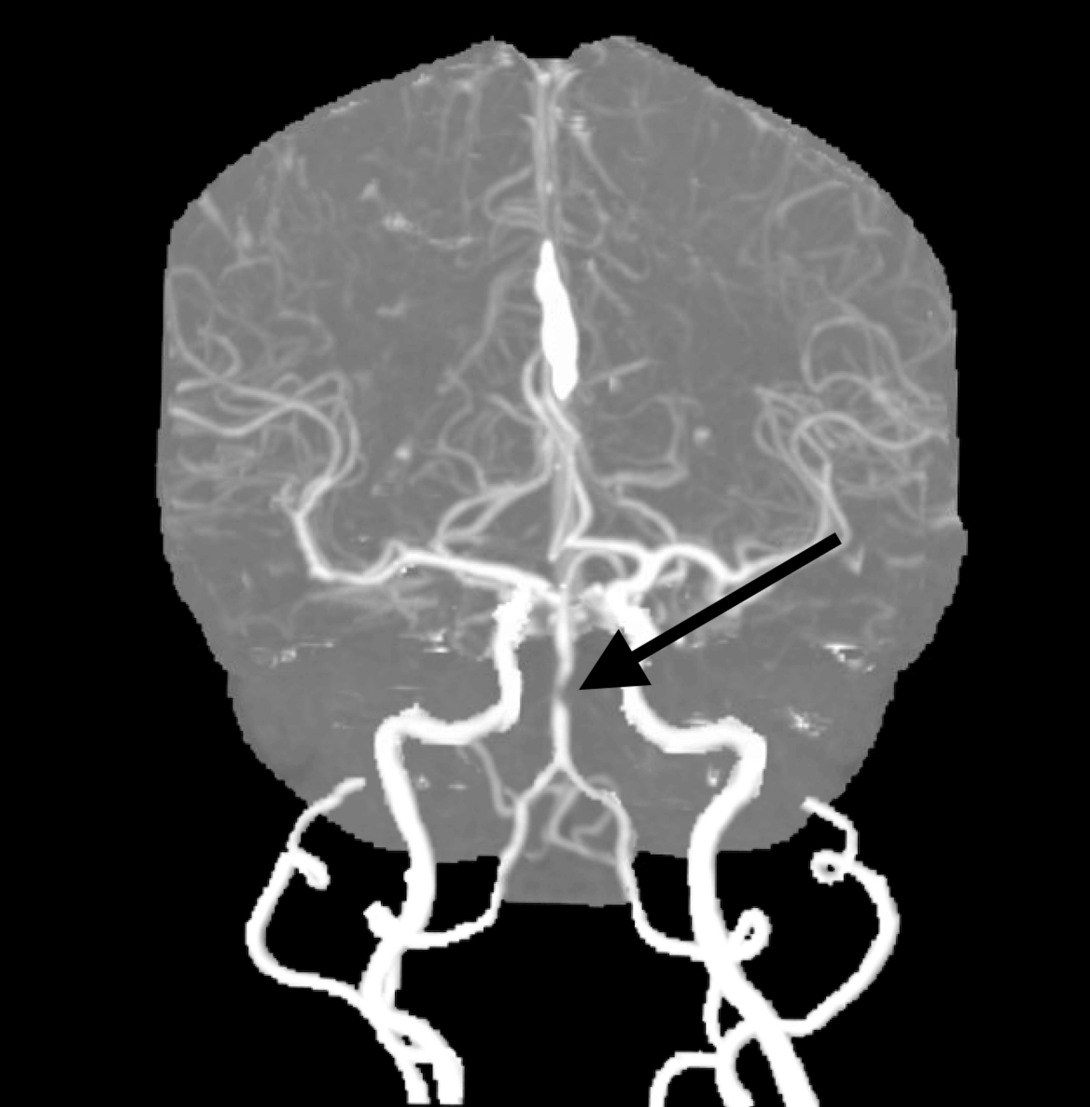
CT angiogram revealed a high-grade mid-basilar stenosis (indicated by the black arrow).

Two years after this initial stroke, the patient presented to the emergency room with right-sided hemiparesis, dizziness, and dysarthria. Soon after presenting to the emergency room, the patient rapidly deteriorated, requiring airway protection, so a complete neurological exam could not be performed. A computed tomography (CT) scan ruled out intracranial hemorrhage, allowing for the administration of IV tenecteplase (TNK). CT angiogram (Figure 2) demonstrated high-grade stenosis of the mid basilar artery, leading to angioplasty with stent placement. MRI was not obtained during this admission due to the patient's clinical deterioration. It is important to note that prior vascular imaging was not available for comparison. Thus, we could not determine if this was a previously unrecognized or new stenosis. The patient was stabilized and cleared for inpatient rehabilitation one week later.

**Figure 3:**
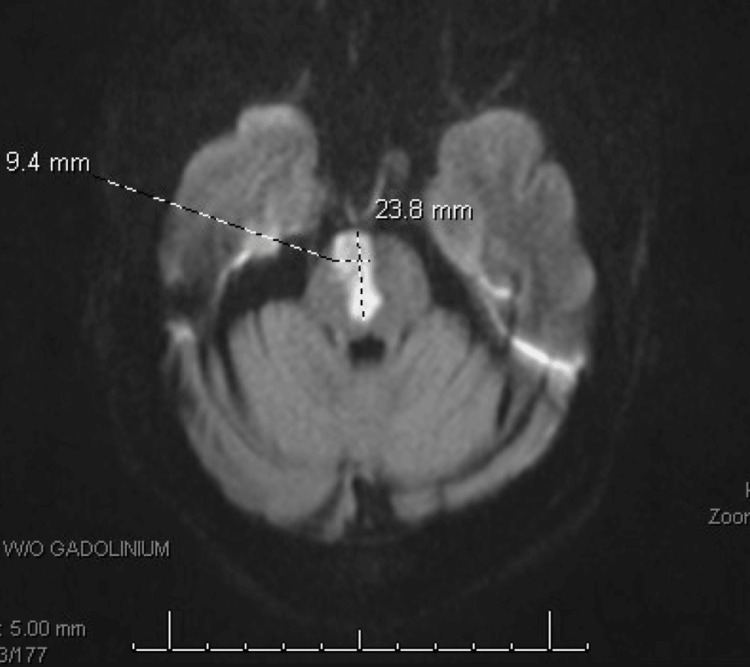
MRI of the brain without contrast demonstrating an acute infarct in the right paramedian pons.

**Figure 4:**
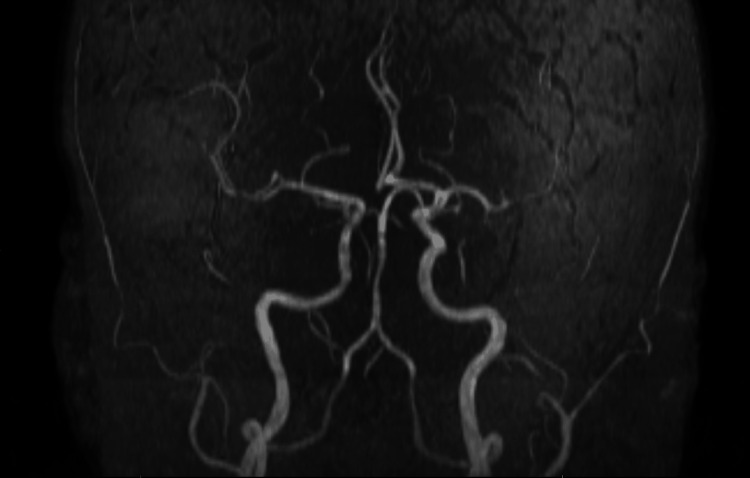
Magnetic resonance angiography (MRA) demonstrating mild basilar artery irregularity, which may be related to prior stenting. There is no evidence of high-grade stenosis or proximal branch occlusion. This suggests that the pontine infarct was not due to large-vessel occlusive disease.

Approximately one month later, the patient presented from inpatient rehab to the internal medicine service due to left-sided weakness and dysarthria. She was transferred to the general neurology floor for a possible new ischemic stroke workup. MRI reported an acute infarct in the right paramedian pons with tiny acute/subacute infarcts in the inferior pons on both sides (Figure 3). There was no evidence of high-grade stenosis of the proximal branches on magnetic resonance angiography (MRA). Mild basilar artery irregularity was noted, possibly related to prior stenting. There was no evidence of stent restenosis or obstruction on MRA (Figure 4). These findings suggest that the pontine infarct was not secondary to a blockage in the large arteries or embolic disease. Moreover, this stroke does not fit the usual diabetic pattern, which usually involves deep supratentorial structures, such as the patient's initial stroke.

A summary of the patient's stroke timeline and imaging findings is presented in Table 1.

**Table 1:** Summary of stroke timeline and diagnostic findings. MRI: magnetic resonance imaging; CTA: computed tomography angiography; MRA: magnetic resonance angiography.

Date	Clinical event	Imaging
2022	Left thalamic lacunar infarct	MRI: Left thalamic infarction
June 2024	Posterior circulation ischemic event	CTA: High-grade mid-basilar artery stenosis
July 2024	Pontine infarction	MRA: Infarct in the right paramedian and inferior pons on both sides. Mild irregularity of the basilar artery, related to prior stenting. Normal flow-related enhancement in the major intracranial arteries with no evidence of proximal branch occlusion or high-grade stenosis.

Due to the unclear etiology of this pontine stroke despite stenting, a hypercoagulable workup was completed. This included studies such as prothrombin time (PT), partial thromboplastin time (PTT), and international normalized ratio (INR). Genetic and thrombophilia testing was also completed and included factor V Leiden, prothrombin G20210A mutation, JAK2 V617F, antiphospholipid antibodies, homocysteine, protein C, protein S, dilute Russell's viper venom time (DRVVT), hexagonal phase phospholipid, and lupus anticoagulant testing. Inflammatory markers such as lactate dehydrogenase, erythrocyte sedimentation rate, and ferritin were also checked. We also ordered vitamin B12/folate levels, immunoglobulin levels, and hemolysis labs (haptoglobin). We also checked tumor markers to rule out cancer as a cause of hypercoagulability. This included alpha fetoprotein, carbohydrate antigen 19-9, cancer antigen 15-3, cancer antigen 125, carcinoembryonic antigen, cancer antigen 27.29, chromogranin A, and monoclonal gammopathy panel. These results were ultimately unremarkable or not clinically contributory except for factor VIII activity, which was 281% (reference range: 50-175%).

## Discussion

This case highlights the diagnostic and management challenges of recurrent ischemic strokes in a patient with both traditional and less common risk factors. The patient presented with three distinct ischemic events over a two-year period. Initially, this presented as a small-vessel left thalamic infarct, likely due to the patient's poorly managed diabetes. This clinical course later progressed to involve major circulation in the form of a high-grade basilar artery stenosis requiring stenting. This was subsequently followed by an acute infarct in the paramedian pons with bilateral acute/subacute infarct in the inferior pons.

The patient's primary risk factor, poorly controlled diabetes, accounts for the initial small-vessel stroke. Additionally, this risk factor can contribute to atherosclerosis in the basilar artery. However, the recurrence of a new pontine stroke despite a patent basilar stent raised doubts about a purely atherosclerotic mechanism. This etiology remaining unclear prompted an evaluation of a hypercoagulable disorder, which included an extensive hypercoagulable workup.

This evaluation revealed an elevated factor VIII activity of 281% (reference range: 50-175%). Factor VIII is a glycoprotein crucial to the extrinsic and intrinsic coagulation pathways [2]. When activated, it promotes thrombin formation and fibrin generation, subsequently creating a hypercoagulable state [2,3]. For venous thrombosis, elevated factor VIII is a well-documented risk factor [4,5]. For example, Kraaijenhagen et al. reported that 57% of patients with recurrent venous thrombosis had factor VIII levels ≥ 150 IU/dL [4]. The Leiden Thrombophilia Study also demonstrated that factor VIII levels >150 IU/dL had a 4.8-fold increased risk of venous thromboembolism [6].

Notably, case reports have been described in the literature highlighting the association of factor VIII with ischemic strokes. For instance, one case presented a cerebral arterial stroke in a 32-year-old female whose only identifiable risk factor was elevated factor VIII activity [7]. Another case described a 40-year-old male who suffered an ischemic stroke, with an elevated factor VIII activity of 408% being the only identifiable risk factor for this event [8]. These cases demonstrate that high factor VIII activity is a possible cause of ischemic strokes, although data are still limited and additional studies are warranted.

The case of this 52-year-old female adds to the growing body of evidence suggesting a link between elevated levels of factor VIII and ischemic strokes. It is important to note that this case reflects an association rather than confirmed causality. This should be interpreted cautiously since factor VIII is an acute-phase reactant and its levels can increase in the presence of inflammation [3,4]. Given the patient's underlying diabetes and vascular disease, inflammation from atherosclerosis or ischemic events can contribute to factor VIII elevation as well [3,4]. This makes it unclear if factor VIII elevation is a cause of thrombosis or a marker for the underlying inflammation [3].

However, the recurrence of ischemic events, in the absence of traditional risk factors such as dyslipidemia, does raise the possibility that factor VIII could increase the risk of a stroke. While this cannot be confirmed, it highlights the importance of considering factor VIII levels in cases of cryptogenic strokes.

## Conclusions

At the time of this submission, routine screening of factor VIII levels is not part of a routine stroke evaluation. Since factor VIII levels vary significantly based on factors such as stress and inflammation, there is still great uncertainty about whether we should check factor VIII levels. However, current literature linking elevated factor VIII levels to venous and arterial thrombotic events suggests that this parameter deserves closer attention in clinical practice. The authors of this case report believe that although universal screening is not warranted, factor VIII testing should be pursued in specific patient populations, particularly those lacking traditional risk factors.
